# An optimisation framework for resource allocation in palliative and end-of-life care

**DOI:** 10.1038/s41598-026-45072-5

**Published:** 2026-04-15

**Authors:** Elizabeth Williams, Syaribah Brice, Daniel Gartner, Paul Harper, Maneesh Kumar, Anthony Byrne

**Affiliations:** 1 School of Mathematics, Cardiff University, Cardiff, CF24 4AG UK; 2 Digital Health & Intelligence, Cardiff and Vale University Health Board, Cardiff, CF14 4XW UK; 3 Aneurin Bevan University Health Board, Newport, NP18 3XQ UK; 4 School of Business and Health, Aalen University, Aalen, 73430 Germany; 5 Cardiff Business School, Cardiff University, Cardiff, CF10 3EU UK; 6 Velindre University NHS Trust, Nantgarw, CF15 7QZ UK; 7 Marie Curie Hospice Cardiff and Vale, Penarth, CF64 3YR UK; 8 Department of Mathematics, Universitas Negeri Malang, Malang, Jawa Timur 65145 Indonesia

**Keywords:** Palliative care, Healthcare optimisation, Resource planning, Data-driven modelling, Health care, Mathematics and computing, Medical research

## Abstract

End-of-life care for frail and elderly patients is frequently characterised by high healthcare utilisation, fragmented service delivery, and limited coordination, resulting in variable quality and excess cost. This study presents a proof-of-concept framework, tested using synthetic data to illustrate potential applications in strategic planning. Few planning approaches integrate patient-level pathways into operational models that balance efficiency with patient-centred outcomes. Optimisation models were developed to support strategic resource planning for frail, elderly, and palliative patients in the final year of life. Two formulations were explored: one minimising overall cost and another aligning demand with available capacity. Patients were stratified into ten representative categories and assigned to structured pathways with varying resource intensities across hospital beds, palliative beds, community nursing, and virtual wards. A synthetic dataset representing plausible twelve-month service trajectories was used to assess model performance. Both models produced feasible allocations that satisfied expected demand within capacity limits. Most patient groups were consistently assigned to dominant pathways, while some shifted depending on the optimisation objective, illustrating trade-offs between cost efficiency and balanced utilisation. Demand intensified in the final months of life but remained manageable under planning assumptions. The modelling framework demonstrates the feasibility of applying optimisation to anticipatory planning, enabling comparison of service configurations and supporting more coordinated, efficient, and patient-centred end-of-life care.

## Introduction

Palliative and End of Life Care (EoL) are critical healthcare services which prioritise the needs, preferences and comfort of patients with chronic or life-threatening conditions^1,2^. These services adopt a holistic approach, addressing not only physical symptoms but also the psychological and social needs of patients and their families. Delivering such comprehensive care requires not only compassionate clinical practice but also effective planning and equitable access to resources across care settings. While EoL care typically refers to the support provided in the final stages of life (i.e “likely to die within the next 12 months”)^3^, palliative care can begin at any point during an illness and focuses on improving quality of life through effective symptom management^1^. Despite their proven benefits, access to these services remains limited globally, hindered by fragmented systems, workforce shortages and inequalities in service provision^2,4^. The palliative and EoL care strategic objectives in UK mirror wider shifts in healthcare policy, from competitive organisational approaches towards an integrated ‘whole systems’ model based on local innovation and delivery (sometimes termed ‘place-based care’), underpinned by national programmes to enable efficient and rapid improvements in seamless patient-focused care^3^. The White Paper in England^5^ and ‘A Healthier Wales’^6^ also recognise that the whole systems model will rely on the power of digital and data to drive transformative integration. The delivery of high-quality, equitable, and cost-effective care during the final stages of life is a persistent challenge for healthcare systems globally^7^. As populations age and the prevalence of complex co-morbidities rises, healthcare systems face increasing demands for coordinated and anticipatory care. However, misaligned care pathways, inadequate integration across providers, and inconsistent planning often result in suboptimal patient experiences and unnecessary utilisation of acute care resources, such as emergency departments and hospital beds^8–10^. Studies have shown that a disproportionate share of healthcare expenditure occurs in the final year of life, much of it driven by unplanned and reactive care episodes that may not reflect patient preferences^11,12^. Effective service delivery requires a multi-disciplinary, integrated approach involving hospital, community and specialist services^13^. In the United Kingdom (UK), palliative and EoL care services are delivered across a network that includes community, primary, secondary, and tertiary care providers^14^. However, coordination across these sectors is often hindered by fragmented information systems, inconsistent resource availability, and operational challenges^15^. Evidence suggests that initiating specialist palliative care earlier in the patient journey can lead to significantly lower use of inpatient and outpatient services, supporting both system efficiency and patient preferences^16^. To address these challenges, healthcare systems are increasingly turning to a range of Operational Research (OR) tools, including optimisation^17^. These tools employ mathematical modelling and computational algorithms to guide strategic resource allocation within constrained systems. By leveraging patient-level data and real-world care pathways, optimisation models can stratify patient needs, forecast service demand and simulate the impact of different care configurations. Techniques such as integer programming and simulation allow decision-makers to design care pathways that are not only cost-efficient but also responsive to patient needs^18,19^. However, previous studies applying these and related optimisation techniques to healthcare resource allocation have often focused on single care settings or specific resource types^20,21^. Few studies capture integrated care pathways across hospital, community, and palliative services, incorporate patient-level heterogeneity, or consider dynamic demand during the final year of life^22^. This study provides a presents a proof-of-concept optimisation framework designed to explore resource allocation strategies for frail, elderly, and palliative care patients, with the potential to inform future redesign of care management using routinely collected healthcare data. By modelling transitions across multiple care settings and stratifying patients into representative types, this study addresses these gaps and provides a framework that can guide strategic planning of resources. Using a synthetic dataset to replicate realistic patterns of healthcare utilisation, the framework enables exploration of care transitions, capacity pressures, and system-level trade-offs within a controlled environment. The two optimisation approaches focus specifically on cost efficiency and demand–capacity balance, providing an operational foundation for future extensions that may incorporate patient-centred outcomes. While patient-centred outcomes are central to high-quality palliative and end-of-life care, operational constraints such as bed availability, workforce capacity, and service configuration fundamentally shape what care can be delivered in practice. This study therefore focuses on the system-level resource allocation problem as a foundational step. Establishing a transparent optimisation framework for cost and demand–capacity management provides the structural basis upon which outcome-based and patient-centred extensions can subsequently be developed. The remainder of this paper is structured as follows: the Methods section details the formulation of the optimisation framework. The Results section presents the outputs of the two optimisation models at both patient and system levels. The Discussion interprets these findings in the context of existing literature, highlights implications for service planning and outlines limitations. Finally, the Conclusions summarise the study’s contributions and its potential for informing real-world planning of palliative and EoL care services.

## Methods

Our research presents a methodological framework for optimising the allocation and timing of healthcare resources for frail, elderly, and palliative patients in the final year of life. As health systems face increasing pressure to deliver coordinated, cost-effective, and patient-centred care, there is a growing need for tools that support proactive service planning across hospital, community, and specialist care settings. We propose two deterministic optimisation models to inform when, where, and how care resources should be deployed across multiple patient subgroups and care pathways. Deterministic models are appropriate in this context because they allow transparent, tractable allocation of known patient demand to available resources, facilitating scenario testing and strategic planning^23^. While deterministic models do not capture stochastic variability in demand, they provide a necessary foundation for evaluating model structure, resource trade-offs, and potential service redesign. Both models operate over a defined planning horizon, divided into discrete time periods (e.g., monthly intervals over a 12-month period). Patients are grouped into illustrative subpopulations based on demographic and clinical characteristics, each representing typical pathways through EoL and palliative care services. In the current implementation, patients are assigned to a single subpopulation for the full planning horizon and do not migrate between patient types. The models share a common structure but differ in their planning objectives:

**Model A: Cost Minimisation Model.** This model supports decision-making by determining the most cost-efficient configuration of resource use that satisfies all patient care needs. It considers the full set of patient demands and identifies when and where care should be provided to minimise total system expenditure, based on unit costs associated with different services (e.g., inpatient stays, community visits, palliative care inputs). While the cost minimisation model prioritises pathways that minimise total expenditure, it fully respects the constraint that all patient demand must be met. In practice, this means that each patient type is assigned to a care pathway, but the chosen pathway balances both cost efficiency and resource feasibility. Where lower-cost pathways are constrained by limited capacity, the model assigns patients to alternative feasible pathways to ensure that demand is satisfied without exceeding available resources.

**Model B: Demand-Capacity Optimisation Model.** This model adopts a resource-focused perspective, enabling planners to determine how to allocate limited care resources, such as beds, staff time, or virtual ward slots, across patient groups and time periods. It identifies allocation strategies that meet the care needs of all patient types while using the smallest feasible volume of resources, supporting efficient and balanced delivery without overextending capacity. By assigning resources according to patient type and pathway requirements, the model provides a transparent and systematic approach to planning care across the system. Both models provide a flexible, scenario-driven environment for evaluating different planning strategies. They support decisions not only about how many resources to allocate, but also when and to whom, helping healthcare systems balance cost, capacity and clinical appropriateness in care delivery. Both models are also formulated with potential for future expansion into a two-stage stochastic optimisation model. In this formulation, decisions made in the first stage (e.g., advanced allocation of community or palliative resources) would influence outcomes in the second stage, where actual patient demand may differ from forecasts. If services are not pre-planned or reserved in the first stage, additional costs, representing reactive, unplanned or more intensive care, are incurred. This structure enables the model to accommodate uncertainty in demand or service availability, supporting more robust and anticipatory planning in dynamic healthcare environments.

### Sets and indices

The sets used within the models provide the foundation for specifying constraints, decision variables, and objective functions. Firstly,$\mathscr {P}$, represents the set of patient types. For example, patient types may be defined using retrospective groupings such as underlying cause of death, in order to characterise distinct patterns of healthcare utilisation in the final year of life. These groupings are used to parameterise representative utilisation profiles within the model. Patients are assumed to belong to a single type for modelling purposes, and no migration between types is permitted. Secondly, $\mathscr {M}$, denotes the set of care pathways, where each pathway represents a possible sequence of care transitions and service use for patients. For example, a care pathway focused on keeping the patient at home for longer would prioritise community-based care and virtual ward support over hospital admissions. Another example of a care pathway may be based on whether a patient received a specialist palliative care referral or not. Thirdly, $\mathscr {N}$, represents the set of resources required to deliver care, such as number of bed days. To illustrate, two individuals classified within the same underlying cause-of-death group may nevertheless experience different care pathways and utilise different volumes of services. Lastly, $\mathscr {T}$, denotes the set of time periods, representing the final months of life. Within our study, we focus on the final 12 months of life, allowing the model to track changes in service needs and resource allocation over time. Together, these sets underpin the optimisation framework used to explore alternative care configurations (Table 1).

**Table 1:** Model sets and indices.

Set	Range	Definition
$\mathscr {P}$	*p *= 1, ..., P	Set of patient types
$\mathscr {M}$	*m *= 1, ..., M	Set of care pathways
$\mathscr {K}$	*k *= 1, ..., K	Set of resource types
$\mathscr {T}$	*t *= 1, ..., 12	Set of time periods

### Parameters

Table 2 displays the parameters used within both deterministic models.

**Table 2:** Model parameters.

Parameters	Definition
$D_{p} \in \mathbb {N}$	Expected number of patients (demand) of patient type $p \in \mathscr {P}$
$C_{k,t} \in \mathbb {N}$	Available capacity of resource type $k \in \mathscr {K}$ at time $t \in \mathscr {T}$
$A_{p,m} \in \mathbb {R}_{\ge 0}$	Cost of assigning a patient type $p \in \mathscr {P}$ to care pathway $m \in \mathscr {M}$
$r^{k,t}_{p,m} \in \mathbb {R}_{\ge 0}$	Amount of resource $k \in \mathscr {K}$ required at time $t \in \mathscr {T}$ assigning a patient of type $p \in \mathscr {P}$ to the pathway $m \in \mathscr {M}$. May be fractional (e.g, expressed as fractions of a full-time equivalent (FTE) nurse).

### Decision variables

The key decision variables in both models are $x_{p,m} \in \mathbb {N}$, representing the number of patients of type *p* assigned to pathway *m*. Patients are assigned to a single care pathway for the entire 12-month planning horizon; pathway assignment does not vary over time. Temporal changes in care needs are instead captured through the time-indexed resource requirements $r^{k,t}_{p,m}$. This variable captures the allocation choices the model must make, determining how patients are distributed across available care options. By varying $x_{p,m}$ within the constraints imposed by patient demand, resource capacities and care requirements, the model identifies allocations that achieve the defined objective.

### Objective functions

Two alternative objective functions are considered to reflect different system goals.

*Model A - Cost Minimisation*

Minimise the total cost of assigning patients to pathways:

1 $$\begin{aligned} \min \sum _{p \in \mathscr {P}}\sum _{m \in \mathscr {M}} A_{p,m}x_{p,m} \end{aligned}$$

*Model B - Demand–Capacity Minimisation*

Minimise the total use of resources across all patient allocations and time periods:

2 $$\begin{aligned} \min \sum _{p \in \mathscr {P}}\sum _{m \in \mathscr {M}}\sum _{k \in \mathscr {K}}\sum _{t \in \mathscr {T}} r^{k,t}_{p,m}x_{p,m} \end{aligned}$$

### Constraints

We consider two optimisation models, each defined by a different objective function: Model A (cost minimisation) and Model B (demand–capacity minimisation). Both objective functions are subject to the following constraints:

1. **Demand Satisfaction**: Each patient type must be fully assigned across available pathways.
  3 $$\begin{aligned} \sum _{m \in \mathscr {M}} x_{p,m} \ge D_{p} \quad \forall p \in \mathscr {P} \end{aligned}$$
2. **Resource Capacity Limits**: Resource use at each time step must not exceed available capacity.
  4 $$\begin{aligned} \sum _{p \in \mathscr {P}}\sum _{m \in \mathscr {M}} r^{k,t}_{p,m} x_{p,m} \le C_{k,t} \quad \forall k \in \mathscr {K}, t \in \mathscr {T} \end{aligned}$$
3. **Non-negativity and Integer Constraints**: Patient assignments must be whole numbers.
  5 $$\begin{aligned} x_{p,m} \in \mathbb {N} \quad \forall p \in \mathscr {P}, m \in \mathscr {M} \end{aligned}$$

### Synthetic data generation

To support feasibility testing and demonstration of the optimisation framework, synthetic patient-level data were generated to reflect plausible patterns of healthcare utilisation during the final year of life. A total of 5,000 patients were randomly assigned to ten synthetic patient types and two care pathways, with care pathway 1 focused on hospital and community nursing resources, and care pathway 2 prioritising virtual wards and community-based care. The ten patient types were constructed solely as illustrative modelling groups to introduce heterogeneity in utilisation patterns; they do not correspond to clinical diagnoses, prognostic categories, or any established taxonomy. Patients were allocated to these types randomly, and the categories should be interpreted as abstract modelling constructs rather than clinically meaningful cohorts. Each patient was assigned to one of the two care pathways. This structure allowed the exploration of trade-offs between different care delivery approaches. In this implementation, the set $\mathscr {K}$ contains four resource types: hospital beds, palliative beds, community nursing hours, and virtual ward beds. The framework is designed to accommodate alternative or more granular patient types, care pathways, and resource categories.

Hospital bed utilisation was informed by published estimates indicating that patients in the last year of life have an average of 2.28 hospital admissions, corresponding to a total of approximately 30.05 bed days, with roughly 10% of patients having no hospital admissions^24^. For each patient, total hospital bed-days were drawn from a normal distribution around this mean, with values then distributed across the 12 months to reflect greater utilisation in the months closest to death.

Palliative bed utilisation was modelled using reported hospice usage, with 6.4% of patients assigned at least one hospice stay and an average length of stay of 18.3 days^25,26^. To introduce temporal realism, individual patient palliative bed-days were distributed across months using a weighted scheme, allowing some usage in earlier months while maintaining higher intensity in the final months of life. This approach captures the variability of patient trajectories and the observed tendency for palliative care to be concentrated near EoL, while still accommodating earlier utilisation for a subset of patients.

Virtual ward and community nurse utilisation were generated using mode-specific weighting factors, reflecting differences in service focus between care pathway 1 (hospital/community-focused) and care pathway 2 (virtual/community-focused)^27,28^. Total utilisation for each patient was drawn from normal distributions informed by typical service use, and then allocated evenly or proportionally across months to create a temporally realistic profile.

Service capacities were defined as 90% of the maximum observed monthly demand for each resource type across the synthetic dataset. This approach ensured that capacity limits were grounded in the upper range of expected utilisation while maintaining sufficient headroom to simulate realistic operational pressures. The 90% threshold reflects common practice in health service modelling, where capacities are typically set below peak demand to capture the impact of system constraints on patient flow and resource allocation. Applying this rule uniformly across all resources (hospital beds, palliative beds, community nursing, and virtual wards) ensured internal consistency and comparability between care pathways. Unit costs for each resource were derived from publicly available sources to provide realistic but generalisable estimates of care costs. Hospital bed use was costed at $\pounds$586.59 per day, consistent with national reference data for general and acute care in England^29^. Specialist palliative care beds were valued at $\pounds$425 per day, reflecting typical hospice-level costs^26^. Community nursing was costed at an hourly rate of $\pounds$25.71^30^. Virtual ward care was assigned a cost of $\pounds$184.38 per day, informed by NHS virtual ward evaluations and commercial data^27^. Costs were applied uniformly across the two care pathways, while utilisation patterns varied by patient type and month before death. These assumptions were used to create a realistic cost structure within the synthetic dataset, supporting feasibility testing of the optimisation model rather than precise economic evaluation.

For each patient, monthly utilisation for all four resources was recorded. Summary statistics were calculated both overall and conditional on patients using a given resource, particularly for low-prevalence resources such as palliative beds. This allowed for reporting of both average utilisation and percentage of patients using the resource per month, providing transparency about zero-inflation patterns common in EoL care.

This synthetic data generation approach ensured that resource use patterns were plausible and consistent with published literature, but values were not derived from real patient data. The primary purpose was to enable robust testing of the optimisation models and demonstration of their ability to allocate resources under controlled, yet realistic, scenarios.

## Results

The optimisation framework was tested on a synthetic dataset designed to approximate care utilisation patterns over the final 12 months of life. These outputs are not intended to represent real-world findings, but rather to demonstrate the model’s structure, assumptions, and capacity to inform resource allocation and service planning decisions in EoL and palliative care settings.

### Synthetic data summary

The synthetic dataset was generated to approximate realistic EoL resource utilisation patterns across two pathways of care delivery. This data was not intended to represent actual patient outcomes, but rather to ensure that the optimisation model was based on plausible healthcare dynamics consistent with published literature.

**Fig. 1:**
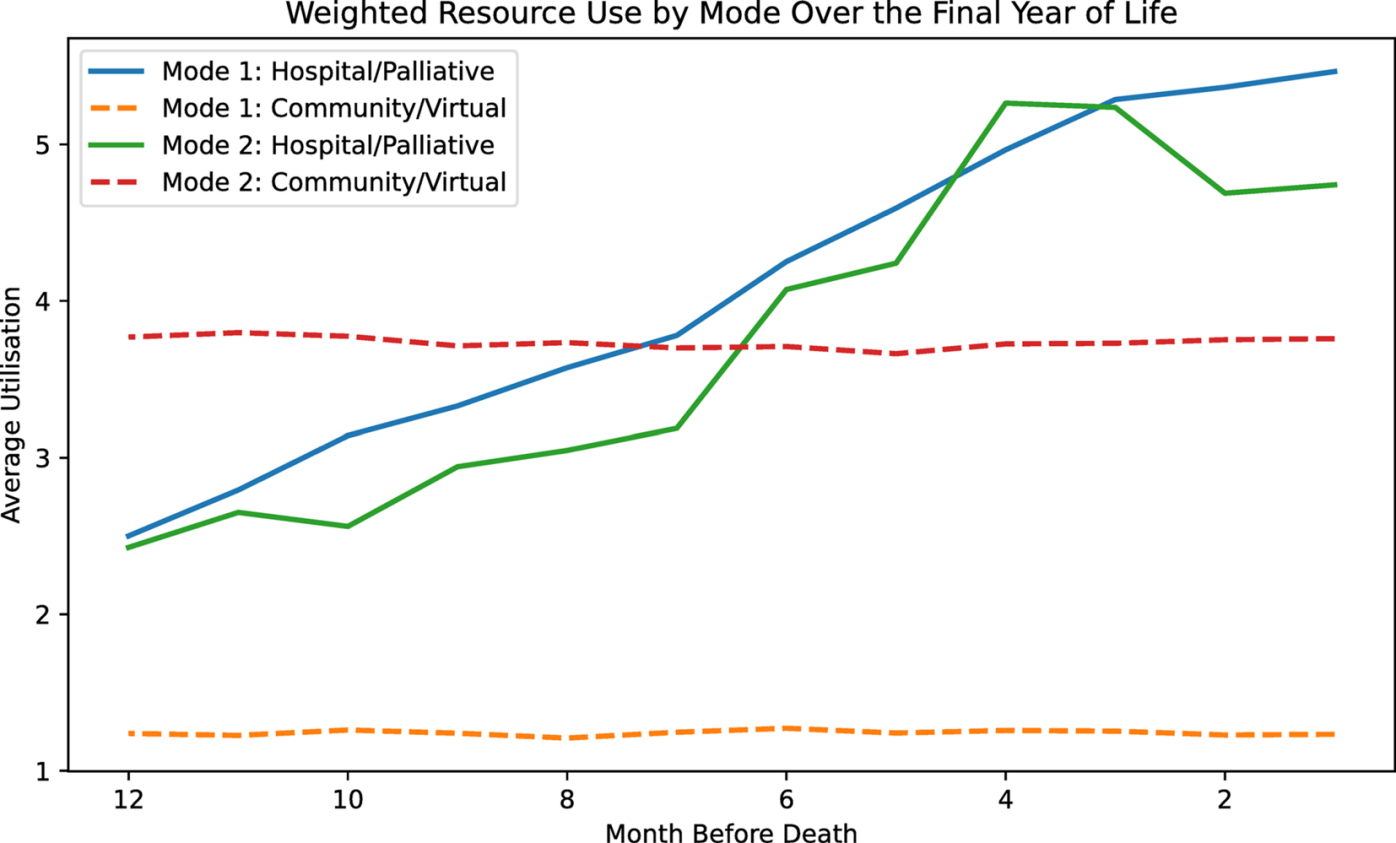
Average resource use by care pathway over the final 12 months of life. Each care pathway is represented by two lines: hospital/palliative utilisation (solid) and community/virtual utilisation (dashed). Care pathway 1 (hospital/palliative) exhibits increasing intensity as death approaches, while care pathway 2 (community/virtual) maintains a steadier use pattern.

Each care pathway was designed to represent different overall pathways of care. Within each care pathway, we separately tracked utilisation of hospital/palliative services (bed days in hospital or palliative units) and community/virtual services (virtual care days and community nursing care hours). Therefore, Fig. 1 shows two lines for each care pathway: one reflecting hospital/palliative resource use, and the other reflecting community/virtual resource use.

**Fig. 2:**
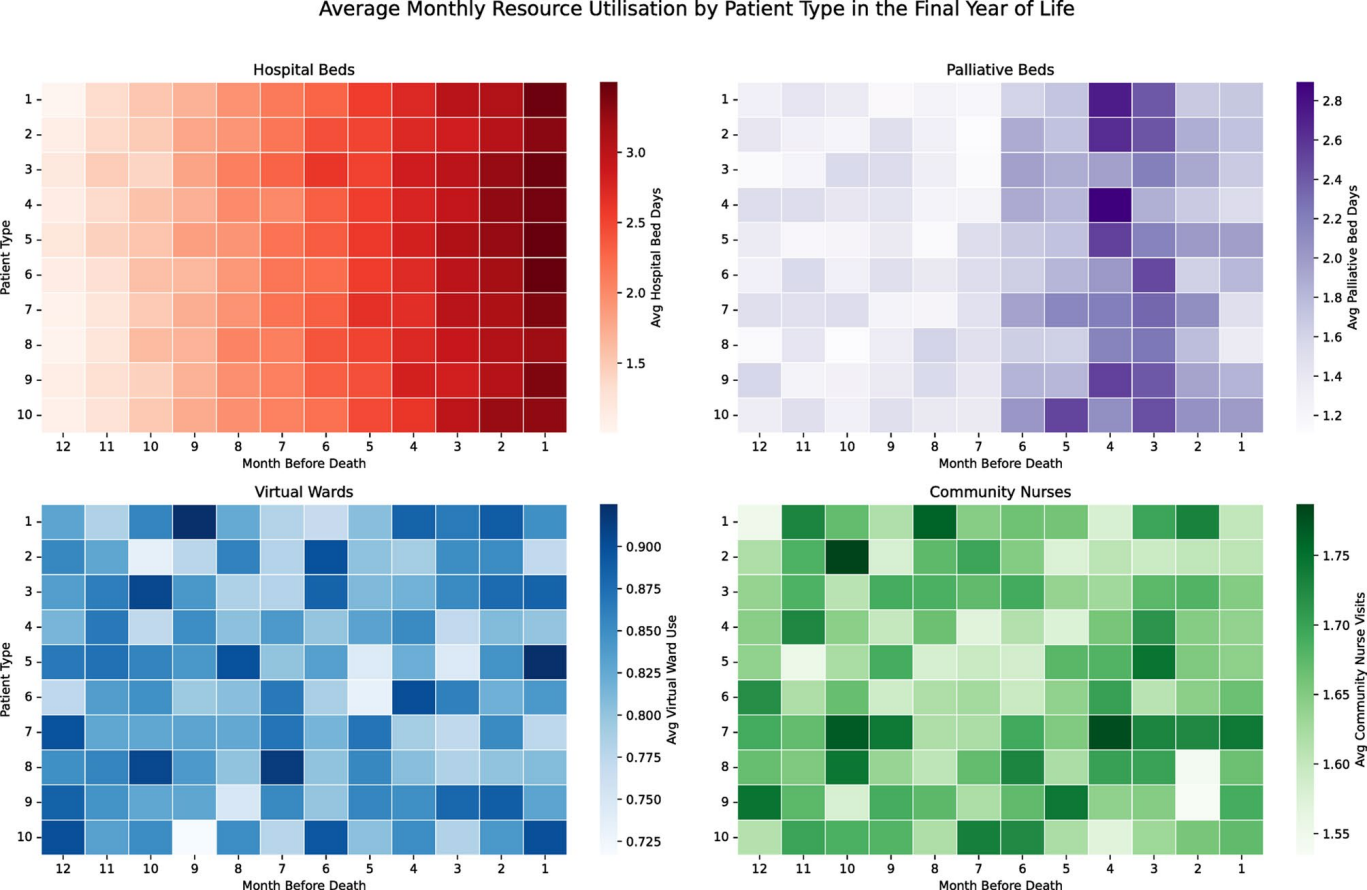
Average monthly resource hospital and palliative bed days over the final 12 months of life.

Figure 2 demonstrates that the synthetic data reproduced expected care trajectories: hospital and palliative bed use increased toward the EoL, while community and virtual care followed more distributed patterns throughout the year. These patterns mirror known service utilisation trends and demonstrate that the generated dataset captures realistic temporal, inter-patient, and inter-care pathway variability.

Overall, this synthetic data approach provides a credible testbed for demonstrating the model’s performance and exploring the feasibility of alternative resource allocation strategies before applying the framework to real-world data.

### Patient pathway assignment

Each of the ten patient types can be assigned to one of two alternative care pathways, each consuming different levels of hospital, palliative, nursing, and virtual ward resources. The optimisation determines the number of patients allocated to each pathway for each patient type. Table 3 presents the assignment of patients to the two care pathways under the two optimisation objectives: Model A (cost minimisation) and Model B (demand–capacity optimisation model). Each row corresponds to one of the ten representative patient types, with entries indicating the number of patients allocated to care pathway 1 or care pathway 2. Each patient type represents a subgroup with distinct care needs, and each care pathway reflects an alternative pathway with differing resource intensities (hospital/palliative versus community/virtual). The optimisation assigns patients to pathways to satisfy total demand while minimising either cost or resource use.

**Table 3:** Assignment of patients to care pathways under Model A (cost minimisation) and Model B (demand–capacity optimisation model).

Patient type	Pathway 1 Model A	Pathway 2 Model A	Pathway 1 Model B	Pathway 2 Model B	Total demand
1	0	533	533	0	533
2	269	201	470	0	470
3	0	513	219	294	513
4	0	486	486	0	486
5	46	461	507	0	507
6	0	513	0	513	513
7	0	480	480	0	480
8	0	516	516	0	516
9	486	0	486	0	486
10	0	496	496	0	496

Both optimisation models maintain total patient demand, ensuring that every patient is allocated to a care pathway. Under Model A, the optimisation model favoured care pathway 2 for most patient types, indicating that the community and virtual-focused pathways were most cost-efficient overall. However, a small subset of patient types were fully or partially assigned to care pathway 1, reflecting cases where the hospital and palliative-oriented pathway was comparatively cheaper despite higher intensity of care. In contrast, Model B shifted the allocation pattern substantially, with most patient types transitioning to care pathway 1. This reflects the trade-off between financial and resource efficiency. While care pathway 2 reduces total costs, care pathway 1 better distributes or minimises the use of constrained capacity across care settings. Overall, the results show that optimisation priorities materially affect allocation outcomes. For most patient groups, one care pathway remains dominant under both objectives, but specific patient types (e.g., 2, 3, 5, 6) demonstrate sensitivity to the optimisation goal. These differences highlight how planning priorities, whether reducing cost or alleviating resource pressures, can meaningfully alter service configuration decisions.

### System level resource utilisation

Following patient-level assignment, the total demand on each resource type was calculated across the 12-month planning horizon. Table 4 summarises the total utilisation of hospital beds, palliative beds, community nursing hours and virtual ward beds under both optimisation objectives, compared with the available capacity. All figures are expressed in bed-days for hospital, palliative and virtual ward beds and nurse-hours for community nursing.

**Table 4:** Total resource use vs available capacity over 12 months under Model A (cost minimisation) and Model B (demand–capacity optimisation model).

Resource type	Capacity (per month)	Model A (Total used)	Model B (Total used)
Hospital Beds	19,200 bed-days	116,042	152,544
Palliative Beds	13,750 bed-days	110,845	89,730
Community Nurses	11,370 hours	133,255	65,404
Virtual Ward Beds	5,760 bed-days	66,853	32,821

The results reveal several key patterns. Under Model A, hospital bed utilisation is lower than under Model B, while community nursing, palliative, and virtual ward resources exhibit contrasting trends. These differences reflect Model A’s prioritisation of lower-cost pathways, which shifts demand away from more resource-intensive options. In contrast, Model B redistributes utilisation to balance pressure across all resources, resulting in higher hospital bed use but lower community nursing and virtual ward demand.

All allocations satisfy total patient demand without exceeding available capacity in any resource category. Over the 12-month horizon, cumulative usage remains below the capacity threshold for each resource, demonstrating that the model successfully distributes patients across care pathways while maintaining feasible resource limits.

**Fig. 3:**
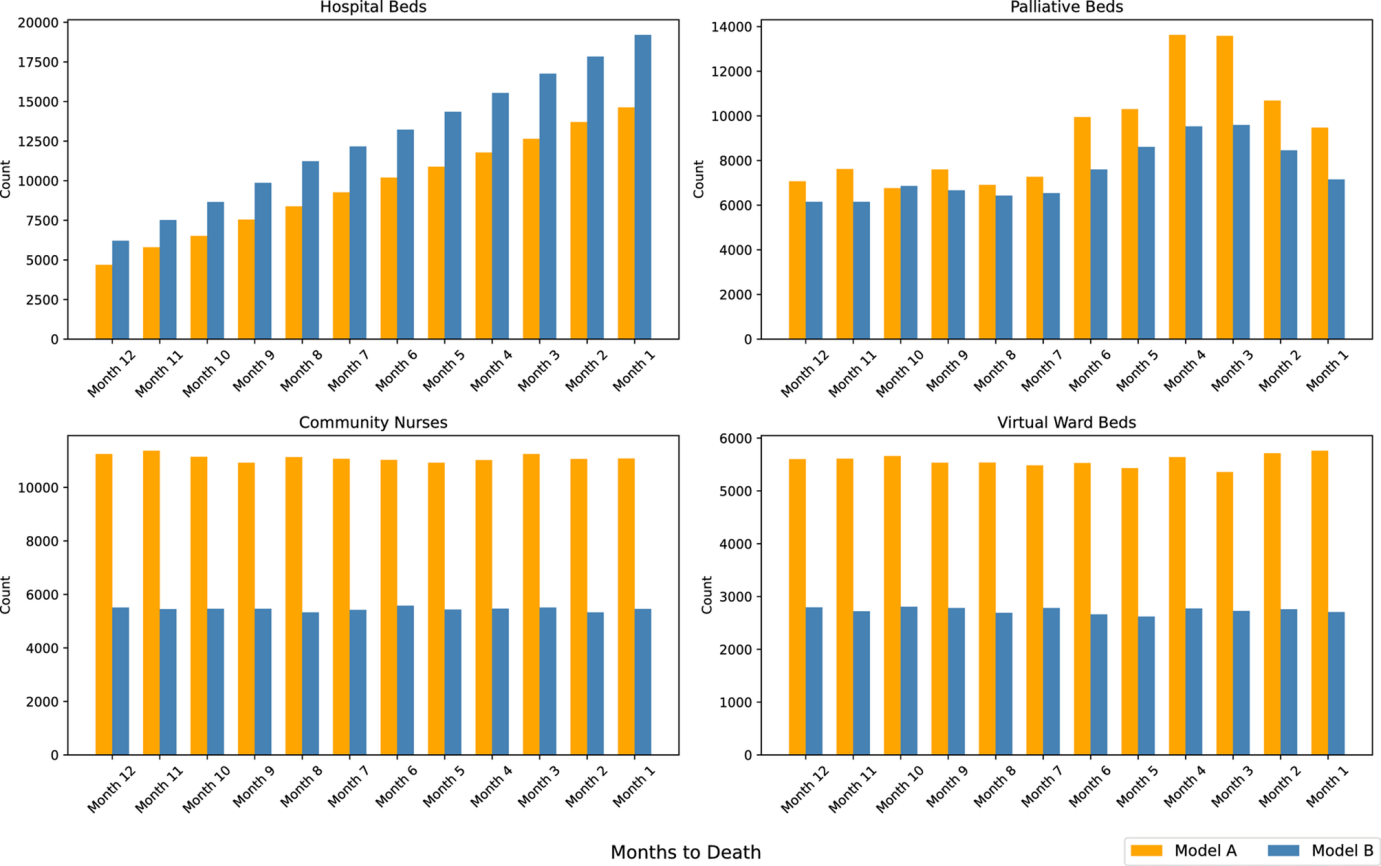
Monthly Resource use by Model for Hospital, Palliative, Community, and Virtual Ward Services.

While these totals provide an overview, they do not capture temporal fluctuations. Monthly utilisation typically rises in the final months of life, especially for hospital and palliative beds, reflecting the higher intensity of care near death (Fig. 3). These peaks highlight the need for anticipatory planning: even when aggregate capacity is sufficient, services must remain responsive to concentrated periods of high demand. Comparisons of total utilisation between Model A and Model B for each resource type indicate statistically significant differences (*p*< 0.05), highlighting that the choice of optimisation objective materially affects allocation patterns. In this synthetic dataset, significance reflects the optimisation constraints rather than inherent stochastic variation.

## Discussion

### Principal results

This study developed and tested an optimisation framework for allocating resources in palliative and EoL care.The framework considered ten representative patient types, two alternative care pathways and four key resource categories (hospital beds, palliative care beds, community nurses and virtual ward beds), over a 12-month horizon prior to death. Two optimisation strategies were investigated: Model A (cost minimisation) and Model B (demand–capacity optimisation model). The framework is flexible and can be readily extended to incorporate alternative or more granular patient groupings, care pathways and resource categories as dictated by data availability and decision context.

Both models generated feasible allocations that satisfied total patient demand without exceeding monthly capacity limits, demonstrating that the framework can successfully manage competing demands across multiple services. However, the models diverged in how patients were distributed across pathways. Some patient types were consistently allocated to one pathway under both strategies, while others were preferentially assigned to an alternative pathway depending on the optimisation objective. These differences led to distinct patterns of resource use: Model A generally prioritised lower-cost pathways, reducing hospital bed demand while increasing reliance on community and virtual care, whereas Model B redistributed patients to balance pressure across all resources, resulting in higher hospital bed utilisation but lower community nursing and virtual ward demand.

Temporal analysis highlighted that resource use intensified in the final months of life across both models. For hospital and palliative beds, demand rose steadily toward the end of the 12-month horizon, reflecting higher care intensity near death. Community nursing and virtual ward demand were more evenly distributed over time but showed notable peaks for some patient types. These monthly dynamics underscore the need for proactive service planning: while aggregate capacity was sufficient, concentrated peaks could strain services if unanticipated.

Statistical comparison of resource utilisation between the two models confirmed that differences were significant across all resource types (*p*< 0.05), demonstrating that the choice of optimisation objective materially affects allocation outcomes. These findings provide insight into how alternative objectives (cost minimisation versus resource balancing) can influence both pathway assignment and system-level utilisation, with implications for service planning and policy in EoL care.

### Comparison with prior work

Previous research on EoL and palliative care planning has primarily focused on descriptive analyses of service utilisation, forecasting demand or modelling single resources such as hospital beds^31,32^. Few studies have applied optimisation methods to explicitly balance cost and capacity across integrated care pathways^33^. In other areas of healthcare, optimisation has been successfully applied to problems such as surgical scheduling^34^, intensive care unit capacity^35^, home health routing^36^ and demand and capacity for elderly & frail patients^37^, demonstrating its potential for improving efficiency in resource-constrained systems. This study extended those approaches into palliative care, where operational decision support tools remain underdeveloped.

This study builds on that body of work by introducing a structured optimisation framework tailored specifically to EoL and palliative care planning. Unlike prior studies that examine single services or forecast demand in isolation, the proposed framework integrates multiple care settings, explicitly models alternative care configurations and jointly considers cost and demand–capacity objectives. By embedding these components within a coherent optimisation structure, this study contributes a formalised approach to EoL care planning, addressing the fragmented nature of existing operational analyses.

### Implications for practice

The findings illustrate how optimisation models can provide actionable insights for service planners, particularly in relation to cost or resource efficiency but also in supporting patient-centred care. By explicitly testing different objectives, healthcare systems can evaluate the trade-offs inherent in service design, such as balancing hospital and community-based pathways to reduce expenditure while maintaining timely access and continuity of care. For example, shifting patients between hospital and community pathways may reduce expenditure but create workforce pressures in community nursing^38^.

The framework also highlights temporal peaks in demand, enabling anticipatory planning to ensure that the right resources are available when needed most, thereby improving the quality and responsiveness of care^39^. Although this study used synthetic data, the modelling approach is directly transferable to real-world datasets, offering potential value for organisations in designing sustainable, efficient, and patient-centred EoL care strategies that balance both system-level efficiency and the needs of patients and families^40^.

This proof-of-concept implementation assumes knowledge of the month of death in order to construct temporally realistic utilisation profiles. We acknowledge that this information is not available prospectively in real-world planning. In practice, a prospective version of the framework would operate on forecasted demand distributions (e.g., expected trajectories for different patient cohorts) rather than exact time-to-death. The steady increase in utilisation observed in the synthetic data reflects known population-level patterns but is not intended to imply perfect foresight at the individual level. Future extensions, such as the stochastic formulation outlined in the Limitations section, would allow uncertainty in both timing and intensity of demand to be incorporated explicitly, enabling more realistic anticipatory planning under imperfect information.

In a real-world setting, the optimisation outputs would be compared against existing service configurations or simple allocation rules to demonstrate the value of the approach. Because this study uses synthetic data, there is no meaningful “current state” against which to benchmark performance. Instead, the models demonstrate feasibility by identifying allocations that satisfy all demand within capacity constraints while minimising either cost or resource use. When applied to real-world datasets, the framework would enable explicit before/after comparisons, such as reductions in cost, avoidance of capacity breaches, or improved balance across care pathways. This would provide a clear reference point for assessing the practical impact of the optimisation.

The study demonstrates the potential real-world application of collaboration between academic researchers, clinical experts, and digital healthcare, with the findings that can be incorporated into a real-world environment via the development of the last year of life population dashboard. Furthermore, this type of project supports clinical data literacy in allowing the healthcare workforce to understand the potential of modelling and simulation to inform practice, to predict and prepare the input data format that informs the models and further data collection.

### Limitations

This study defines the patient cohort retrospectively based on the last 12 months of life. We acknowledge that in practice, individual prognosis is uncertain, and care must be delivered without knowing who will die within a specific time frame. As such, this approach does not aim to predict individual outcomes or evaluate interventions at the patient level. Instead, it uses population-level, retrospective data to model system-level resource allocation, explore trade-offs across care pathways, and assess the potential impact of alternative planning strategies. While LYOL framing cannot directly inform real-time clinical decisions for individual patients, it provides insight into the behaviour of the care system under known patterns of demand, allowing planners to anticipate resource pressures, test service configurations, and inform strategic decision-making aligned with UK NHS and government policy on integrated care. Future extensions of this framework could incorporate stochastic modelling and predictive approaches to better account for uncertainty in individual prognosis.

The results presented here are based on synthetic data and should be interpreted as a proof-of-concept rather than representative of real-world utilisation. The data were generated to reflect structured patterns of service utilisation and resource intensity over time, but they do not capture the full variability observed in real-world patient pathways, workforce availability, or service delivery models. While the framework assumes fixed capacity and cost structures, NHS capacity is generally stable, so this assumption is unlikely to substantially affect system-level insights. The model does not incorporate stochastic fluctuations in demand, geographical variation, or patient preferences.

An additional limitation concerns the treatment of unit costs. In this proof-of-concept framework, costs were represented using fixed average values to support transparency and feasibility testing. In practice, hospital and hospice costs can exhibit wide, non-normal distributions with substantial overlap, driven by patient complexity, unplanned events, and variability in care intensity^41^.Consequently, relative costs are not known deterministically in advance and may differ between patients and episodes. While average costs do not capture this uncertainty, they remain appropriate for demonstrating model behaviour and structural trade-offs. Future work will incorporate stochastic or distributional cost modelling to support robust operational decision-making.

Consequently, the findings demonstrate the feasibility and potential applications of the optimisation framework, rather than providing policy-ready recommendations. The developed models focused on the utilisation of services and cost implications without considering the patients’ behaviour. In palliative and EoL care, considering the patient’s preference is important to ensure the patient’s dignity and comfort. Future work should consider a more complex model that incorporates the patient’s preference as well as patient-reported outcomes.

### Conclusions

This study demonstrates that optimisation modelling can be a powerful tool for planning EoL care, allowing healthcare systems to balance multiple resources while meeting patient demand. By explicitly testing different objectives, the approach highlights trade-offs between cost, resource utilisation, and pathway allocation, and reveals temporal peaks that require anticipatory planning. Although based on structured synthetic data, the framework is readily transferable to real-world datasets and could support the design of efficient, sustainable, and patient-centred care strategies. Future work should incorporate stochastic demand, geographical variation, and patient preferences to fully capture the complexity of real-world service planning.

## Data Availability

Data underpinning this publication can be found in the Cardiff University Research Data Repository at 10.17035/cardiff.31914213.
